# How HSCs Colonize and Expand in the Fetal Niche of the Vertebrate Embryo: An Evolutionary Perspective

**DOI:** 10.3389/fcell.2019.00034

**Published:** 2019-03-12

**Authors:** Christopher B. Mahony, Julien Y. Bertrand

**Affiliations:** Department of Pathology and Immunology, Faculty of Medicine, CMU, University of Geneva, Geneva, Switzerland

**Keywords:** zebrafish, mammals, CHT, fetal liver, hematopoietic (stem) cells, caudal hematopoietic tissue, microenvironment

## Abstract

Rare hematopoietic stem cells (HSCs) can self-renew, establish the entire blood system and represent the basis of regenerative medicine applied to hematological disorders. Clinical use of HSCs is however limited by their inefficient expansion *ex vivo*, creating a need to further understand HSC expansion *in vivo*. After embryonic HSCs are born from the hemogenic endothelium, they migrate to the embryonic/fetal niche, where the future adult HSC pool is established by considerable expansion. This takes place at different anatomical sites and is controlled by numerous signals. HSCs then migrate to their adult niche, where they are maintained throughout adulthood. Exactly how HSC expansion is controlled during embryogenesis remains to be characterized and is an important step to improve the therapeutic use of HSCs. We will review the current knowledge of HSC expansion in the different fetal niches across several model organisms and highlight possible clinical applications.

## Introduction

Hematopoiesis is a highly conserved process across many organisms that culminates with the emergence of hematopoietic stem cells (HSCs). In zebrafish and mammals, hematopoiesis initiates with the emergence of primitive myeloid and erythroid cells (Palis et al., 2001; Bertrand et al., 2005; Palis, 2014; McGrath et al., 2015). Similar cells, prohemocytes, are also detected in drosophila larvae that give rise to plasmatocytes (macrophage-like cells) and crystal cells (platelet-like cells) (Lebestky et al., 2000). Primitive myeloid and erythroid cells are also detected in xenopus embryos (Ciau-Uitz et al., 2014). Following this, definitive hematopoiesis then occurs in two distinct waves in vertebrates. The first wave is characterized by the transient erythro-myeloid precursors (EMPs) that arise in the yolk sac in mice and humans (Bertrand et al., 2005; McGrath et al., 2015), the posterior blood island in zebrafish (Bertrand et al., 2007) and the posterior-lateral ventral blood island in xenopus (Ciau-Uitz et al., 2014). The appearance of EMPs in chicken embryos remains to be determined. The second wave consists of HSC specification from the aortic hemogenic endothelium by the highly conserved process of endothelial-to-hematopoietic transition (EHT). The formation of the hemogenic endothelium requires the correct balance of extrinsic and intrinsic factors to initiate the expression of specific transcription factors, such as *runx1* and *gata2*. During mammalian and avian development, HSC specification occurs in the aorta-gonads-mesonephros (AGM) region where they form intra-aortic clusters (Jaffredo et al., 2000; Bollerot et al., 2005a,b; Zovein et al., 2008; Chen et al., 2009; Boisset et al., 2010) between embryonic (E) day 9.5 and 11.5 in mice, between E26 and E40 in humans and between E3-4 in chickens. During zebrafish development, HSCs emerge between 32 to 60 hours post fertilization (hpf), from the hemogenic endothelium in the dorsal aortal (Bertrand et al., 2010; Kissa and Herbomel, 2010), a process that requires inflammatory cytokines produced by neutrophils (Espin-Palazon et al., 2014) and extracellular matrix (ECM) degradation by macrophages to allow HSCs to enter circulation (Travnickova et al., 2015). HSCs are first detected in the ventral blood island and then later in the dorsal lateral plate mesoderm in xenopus (Ciau-Uitz et al., 2000) and transient cells with HSC characteristics are closely associated to the cardiac tube in drosophila (Dey et al., 2016).

In all these organisms, the initial specification from endothelial cells (ECs) results in a limited number of HSCs that must mature and expand. This is achieved by migrating through different niches, each in distinct anatomical locations that contain specific microenvironments. The first niche that expands HSCs in mouse, humans and xenopus is the fetal liver (FL) (Ema and Nakauchi, 2000; Ciau-Uitz et al., 2014) before they migrate to the bone marrow (BM). In contrast, zebrafish HSCs expand in the caudal hematopoietic tissue (CHT) (Tamplin et al., 2015) and then migrate to the kidney marrow (KM) and chicken HSCs expand in the para-aortic foci (PAF) before seeding the BM (Dunon and Imhof, 2000; Jaffredo et al., 2000; Bollerot et al., 2005a,b). This process is different in drosophila where an initial wave of HSCs (derived from head mesoderm) arises early during larvae development, followed by a second wave of HSCs found in the lymph gland (Lebestky et al., 2000; Dey et al., 2016). HSCs are then seeded in hematopoietic clusters in the dorsal abdomen of adult drosophila (Ghosh et al., 2015; Dey et al., 2016).

Mouse and zebrafish studies have shown that HSCs physically interact with ECs that promote their proliferation in the fetal niche (Tamplin et al., 2015; Khan et al., 2016). We, and others, have shown that this expansion depends on the expression of several cytokines produced by stromal cells and caudal ECs (cECs) (Tamplin et al., 2015; Mahony et al., 2016, 2018). Additional signals are also important for their expansion, which will be discussed in this review. This embryonic expansion is an essential step in the formation of the adult HSC pool and the correct maturation of HSCs.

The self-renewing and multipotent properties of HSCs make these cells an excellent target for regenerative medicine protocols (Cavazzana-Calvo et al., 2000; Walasek et al., 2012; Aiuti et al., 2013). Many therapies currently make use of *ex vivo* expansion of autologous human HSCs, using a cytokine cocktail, but always with limited efficiency (Petzer et al., 1996; Cavazzana-Calvo et al., 2000; Schuster et al., 2012). Therefore a better understanding of the different combinations of cytokines present in the niche along with the additional mechanisms and signaling pathways that normally expand HSCs is required to improve clinical treatment of a range of hematopoietic diseases. Here, we review the recent literature that describes the extrinsic signals important for HSC homing, expansion and finally release from the embryonic niche across zebrafish, xenopus, chicken, and mammals. The anatomical sites where hematopoiesis occurs in these organisms are summarized in Table 1. We will then briefly discuss the possible clinical implications of this current knowledge.

**Table 1:** Summary of anatomical sites of hematopoiesis in the mentioned species.

Species	HSC emergence	HSC expansion	Adult hematopoiesis
Human	AGM	Fetal liver	Bone marrow
Mouse	AGM	Fetal liver	Bone marrow
Chicken	AGM	PAF/YS	Bone marrow
Xenopus	VBI/DLP	Fetal liver	Bone marrow
Zebrafish	AGM	CHT	Kidney marrow
Drosophila	Lymph node	Lymph node	Hematopoietic hubs

*AGM, aorta, gonads, mesonephros. VBI/DLP, ventral blood island/dorsal lateral plate mesoderm. PAF, para aortic foci. YS, yolk sac. CHT, Caudal hematopoietic tissue.*

## HSC Emergence and Homing to the Embryonic Niche

### Mammals

Mammalian HSCs are produced from the floor of the embryonic aorta in the AGM region (Zovein et al., 2008; Chen et al., 2009; Ivanovs et al., 2011) but also in the vitelline and umbilical arteries (de Bruijn et al., 2000). Additionally, human and murine studies have detected HSCs in the placenta that arise independently from and in parallel with the HSCs from the AGM (Gekas et al., 2005; Ottersbach and Dzierzak, 2005; Rhodes et al., 2008; Gekas et al., 2010). However it remains unknown what contribution placenta-derived HSCs make to the adult stem cell pool. HSCs then colonize the FL from E11 in mice and E28 in humans, mainly in response to CXCL12 (Chou and Lodish, 2010). CXCL12, released from ECs, stromal cells and mesenchymal progenitors, is well characterized for its role in HSC homing, retention and survival in the niche (Ara et al., 2003; Christensen et al., 2004; Sugiyama et al., 2006; Sawitza et al., 2009; Greenbaum et al., 2013). CXCL12 enhances migration of FL-HSCs in combination with stem cell factor (SCF), when compared to BM-HSCs (Christensen et al., 2004). Furthermore, mice lacking CXCL12 or its receptor (CXCR4) display normal FL hematopoiesis but aberrant spleen and BM colonization, suggesting that specific and distinct signaling environments attract and maintain HSCs (Nagasawa et al., 1996; Ara et al., 2003). In the FL, HSCs are found closely associated with ECs and stromal cells that promote HSC expansion (Tamplin et al., 2015; Khan et al., 2016).

In addition to cytokine secretion, a direct contact between the different cells within the FL and hematopoietic progenitors is also important to maintain and expand HSCs (Nanno et al., 1994; Corlu et al., 1998). HSCs express a number of integrins and adhesion receptors that are critical for the correct trafficking of HSCs to the FL and could mediate cell contact. For example, VE-Cadherin (CD144), α2b-integrin (CD41), β1-integrin (CD29), cKIT and CXCR4 are well established trafficking molecules expressed by HSCs and play a key role in HSC guidance to the fetal niche (Mazo et al., 2011).

Further studies have demonstrated that umbilical HSCs have a higher affinity to adhere to adult BM than embryonic HSCs, which is due to a specific shift in the expression of specific integrins by HSCs. This suggests that integrin expression is required during development to mediate homing to specific niches (Roy and Verfaillie, 1999). Integrins (mainly α4-integrin) are implicated in mediating HSC interaction with the vascular niche in the BM (Mazo et al., 1998), and their inhibition mobilizes HSCs from the FL (Kim et al., 2016). Human endothelium-derived HSCs also express and use a myeloid adhesion factor, glycosylphosphatidylinositol-anchored surface protein (GPI-80; also known as *Vanin-2*, or *VNN2*) (Prashad et al., 2015), to facilitate their migration and expansion in the fetal niche.

Several ECM, cell adhesion and cytoskeleton pathways are enriched in the AFT024 murine FL fibroblast-derived stromal cell line [a cell line that supports HSC expansion *in vitro* (Nolta et al., 2002)] and within HSCs, permitting HSC migration and anchoring to their niches (Charbord and Moore, 2005).

### Chicken

Definitive chicken hematopoiesis is initiated at E3-4 by the emergence of intra-aortic clusters of HSCs derived from endothelium (as seen in mammals) (Jaffredo et al., 2000; Bollerot et al., 2005a,b; Yvernogeau and Robin, 2017). HSCs then migrate to the neighboring mesenchyme, ventral to the aorta and located in the PAFs, that support the development of CD45^+^ cells (Cormier, 1993; Geerts et al., 1993), such as myeloerythroid progenitor cells and immature thymic precursors (that have not yet undergone T-cell receptor rearrangements) (Lampisuo et al., 1999; Jaffredo et al., 2000; Liippo et al., 2000; Saynajakangas et al., 2009). An additional site of embryonic hematopoiesis includes the yolk sac, which also contributes to the expansion and maturation of erythroid and myeloid cells (Guedes et al., 2014). However, the homing signals to the chicken PAFs remain unidentified. Although little is known about the microenvironment that would support HSCs in the chicken PAFs, differential expression of integrins may play an important role in supporting HSCs (Corbel, 2002).

### Xenopus

Fate-mapping and grafting experiments showed that *bona fide* HSCs are generated in the dorsal lateral plate (DLP), the equivalent of the mammalian AGM (Turpen et al., 1981; Maeno et al., 1985; Ciau-Uitz et al., 2000; Clements and Traver, 2013). In larval stages, DLP-derived HSCs reach maturity and seed the FL where they produce erythrocytes that will replace embryonic primitive erythrocytes. The FL is the main site of HSC expansion and differentiation during embryogenesis, i.e., before metamorphosis (Chen and Turpen, 1995). Classical studies made use of kidney and liver sections from bullfrog tadpoles to reveal hematopoietic microenvironments, supporting red blood cell development (Broyles et al., 1981). After metamorphosis, the majority of the blood cells are DLP-derived (Ciau-Uitz et al., 2014).

### Zebrafish

During zebrafish development, *gata2b* (the earliest hemogenic endothelium marker) is expressed in ECs in the floor of the dorsal aorta (Butko et al., 2015). HSCs are then specified through the expression of *runx1* and *cmyb*, and can be observed undergoing EHT from the floor of the dorsal aorta between 32–60 hpf (Bertrand et al., 2010; Kissa and Herbomel, 2010; Mahony et al., 2018). Contrary to mammals, zebrafish HSCs then migrate toward the vein, where they enter circulation to migrate to the CHT (Bertrand et al., 2010). Within the CHT they considerably expand between 3 and 4 days post fertilization (dpf) (Murayama et al., 2006; Tamplin et al., 2015; Mahony et al., 2016; Staal et al., 2016). HSCs migrate to the CHT embryonic niche in response to *cxcl12a*, expressed from stromal cells (Tamplin et al., 2015). Further zebrafish studies have identified *klf6a* as an important transcription factor that directly regulates *ccl25b* expression in ECs (Xue et al., 2015, 2017). Xue et al. (2017) demonstrated that *ccl25b* is expressed in the CHT at 48hpf and is an important cytokine for HSC chemoattraction to and expansion within the CHT niche. These results were further corroborated by the *ex vivo* culture of murine HSCs in the presence of *Ccl21* (murine ortholog of *ccl25b*) that enhanced HSC expansion through activation of its receptor, *Ccr7* (Xue et al., 2017). Upon arrival in the CHT niche, VCAM^+^ macrophages are also required to direct HSCs (through binding to α*4-integrin* expressed by HSCs) toward venous capillaries and retain them in their embryonic niche (Li et al., 2018).

## Non-Cell-Autonomous Mediators of Hsc Expansion in the Embryonic Niche

The HSC pool first undergoes expansion shortly after HSC emergence from the AGM (Taoudi et al., 2008; Rybtsov et al., 2016), before migrating to their fetal niche. The number of HSCs then greatly expands to around 38 times their original number, peaking at around E14 in mice and ceasing around 2–4 days postnatal (Morrison et al., 1995; Ema and Nakauchi, 2000; Baumann et al., 2004; Lessard et al., 2004; Chen et al., 2009; Payushina, 2012). Therefore, fully characterizing the different cells and environmental cues that expand HSCs in different organisms is required to improve the currently limited regenerative therapies. We will hereafter describe the different elements of the microenvironment that contribute to this expansion, across the vertebrate phylum.

### Stromal Cells

In the mouse embryo, HSCs are closely associated with Nestin^+^ periportal stromal cells that express many HSC expansion factors, such as *angptl2* (Khan et al., 2016). Many different supportive stromal cell lines also have been derived from the mouse FL, such as AFT024, that support HSCs *in vitro* (Nolta et al., 2002), and the KM3 cell line, that supports human embryonic stem cells (Hu et al., 2012). The analysis of the AFT024 cell line revealed an enrichment in secreted factors such as insulin like growth factor, SCF, angiopoietin-3, Wnts and Ephrin2a that support HSCs (Charbord and Moore, 2005).

A subtype of stromal cells, stellate cells, are fat storing hepatic sinusoid cells that appear around E10-11 in the mouse embryo and express a number of cytokines, ECM and adhesion molecules (Ramadori and Saile, 2002; Tan et al., 2017). Stellate cells are *Desmin*-positive and are found in close proximity to HSCs (Kiassov et al., 1995). These cells express many different supportive hematopoietic cytokines such as *OSM*, *Csf1*, *THPO*, *EPO, Igf1, SCF/KitL* and *Cxcl12* (Fujio et al., 1994; Kubota et al., 2007; Tan et al., 2017). Stellate cells also express *VCAM*, *fibronectin1, vitronectin1*, *Lamb1-1* (Laminin-b1-1) and *Lamc1* (Laminin-c1) (Kubota et al., 2007; Tan et al., 2017). *In vitro*, adult hepatic stellate cells can maintain HSCs in a similar manner to BM mesenchymal stem cells (Kordes et al., 2013). Stellate cells may therefore play an important role in maintaining the liver microenvironment and expanding the HSC pool.

Recent studies have also focused on zebrafish stromal cells, derived from the somites (Murayama et al., 2015). Isolated CHT zebrafish stromal cells [caudal hematopoietic embryonic stromal tissue (CHEST)] from 3dpf embryos express a range of hematopoietic cytokines, some of which are not present in isolated kidney cells from adult fish (such as *gcsfb*, *il11a*, *il11b*, and *fgf21*). Furthermore CHEST cells were able to expand cultured HSCs and stimulate HSC differentiation *in vitro* (Wolf et al., 2017). The development of these zebrafish stromal cell lines is an important step and represents a valuable tool to study hematopoiesis in this model. Indeed, by comparing the transcriptome of CHEST, ZKS (zebrafish kidney stromal cells) and ZEST (zebrafish embryonic stromal trunk cells), Berrun and colleagues highlighted the hematopoietic role of *isthmin 1* (*ism1*), a secreted protein required for HSC development as well as erythro-myeloid differentiation (Berrun et al., 2018).

In addition to hematopoietic cytokines, lipid metabolism is also important for HSC expansion and development during embryogenesis. This was recently demonstrated through the study of *lipoprotein lipase* (*lpl*), an enzyme expressed by stromal and/or ECs in the CHT, and required for fatty acid metabolism. Both *lpl* and its cofactor *apolipoprotein c2* (*apoc2*) controlled the release of an essential fatty acid, (Docosahexaenoic acid), which was identified as a novel HSC expansion factor (Liu et al., 2018). This study highlighted an additional possible pathway important for improving HSC expansion *ex vivo*.

The anterior lobe of the drosophila lymph gland consists of a medullary zone (MZ), a cortical zone (CZ) and the posterior signaling center (PSC). Prohemocytes are located in the MZ and give rise to mature hemocytes, plasmatocytes, crystal cells and rare lamellocytes, in response to immune challenge (Evans et al., 2003). These progeny cells will then colonize the CZ. The drosophila PSC is an important signaling niche that controls blood cell production and maturation and has been associated to mammalian stromal cells (Lebestky et al., 2003). The PSC is a source of Hedgehog signal that activates the zinc finger transcription factor *Cubitus interruptus* (Ci) (homolog of the vertebrate Gli proteins) in cells located in the MZ to maintain quiescence of blood progenitors and fine tune differentiation upon immune challenge, independently of the EBF transcription factor Col within blood progenitors (Mandal et al., 2007; Benmimoun et al., 2015a,b; Pennetier et al., 2012). Further studies have suggested that the PSC may interact with nearby cells directly though thin processes that extend into the MZ (Mandal et al., 2007). Additional signals that affect PSC signaling and HSC maintenance include odorants that stimulate γ-aminobutyric acid release from the brain and drosophila *insulin-like growth factor 2* by adipocytes (Yu et al., 2018).

### Endothelial Cells

HSCs arrive in the zebrafish CHT niche and progressively colonize this tissue from 48 hpf until 80 hpf (Tamplin et al., 2015). Although some HSCs are still present at 96 hpf, the majority have proliferated and left the CHT niche (Mahony et al., 2016). To accommodate HSCs within the CHT, the vascular niche is remodeled to improve stem cell seeding, a process that is controlled by *cxcr1* (Blaser et al., 2017). Upon arrival to the CHT niche, HSCs trigger a “cuddling” behavior from caudal endothelial cells (cECs). This cuddling allows the cECs to maintain close proximity between an HSC and a stromal cell, which induces HSC proliferation (Tamplin et al., 2015). The cuddled HSC then undergoes cell division where either both daughter cells will leave the niche, one will leave and the one most proximal to the stromal cell will stay or both will stay. Strikingly, pharmacological stimulation of HSC niche engraftment leads to an overall increase in the number of adult stem cells, as shown by lineage tracing (Tamplin et al., 2015). This indicates the contribution of HSC embryonic niche engraftment to increasing the adult stem cell pool size, and highlights the importance of fully understanding the cytokines expressed by ECs that expand HSCs.

Direct physical contacts between HSCs and ECs have been described in the mouse embryo, similarly to the zebrafish CHT (Tamplin et al., 2015), and may be mediated by *E*-selectin and VCAM1 (Schweitzer et al., 1996; Wittig et al., 2010). Murine FL-HSCs express the *endothelial protein C receptor* that can bind the activated *protein C*. This induces protease-activated receptor 1 signaling which inhibits apoptosis and maintains self-renewal activity (Iwasaki et al., 2010). In contrast to murine HSCs, human HSCs express endothelial protein C receptor in the FL, but lose its expression once they have migrated to the BM (Subramaniam et al., 2018).

Fetal liver ECs also support hematopoiesis *ex vivo* and promote HSC differentiation (Ohneda and Bautch, 1997; Wittig et al., 2010). FL ECs support hematopoiesis through the expression SCF/KitL that is normally membrane-bound. The conversion of this cytokine to its soluble form occurs under the control of *MMP9*, which transcription is regulated by Ezh2, a transcriptional repressor expressed by FL ECs. This process was only described in the context of erythropoiesis (Neo et al., 2018) but might be relevant for HSC expansion.

These studies underscore the importance of ECs in expanding HSCs in the zebrafish CHT and the FL in mammals. However, many other cell types support HSC expansion during embryogenesis.

### HSC Regulation by Their Hematopoietic Progeny

Regulation of HSC expansion by their progeny has been described in the drosophila lymph gland. PDGF and VEGF-related factor- 1 (Pvf1) is produced by the PSC and activates its receptor (PVR) on differentiated cells in the cortical zone. This activates JAK/STAT signaling and consequently controls the expression and secretion of adenosine deaminase Growth Factor-A (Adgf-A) from differentiated cells. This enzyme converts adenosine into inosine that promotes progenitor maintenance, through the PKA intracellular pathway (Mondal et al., 2011). Similarly in mammals, the placental microenvironment supports hematopoiesis through PDGF-B expression to overall inhibit HSC differentiation (Chhabra et al., 2012). Drosophila *bip1* (*bric-à-brac interacting protein 1*) and *Nucleoporin 98 (Nup98)* are additional genes identified in controlling blood progenitor proliferation that control PVR expression (Mondal et al., 2014). The negative feedback of differentiated cells into HSCs has been evidenced in the adult mouse BM, but remains undocumented in the FL.

### Mammalian Hepatocytes

Hepatocytes contribute to most of the liver mass and therefore also play a role in the HSC niche. The analysis of human FL samples has revealed the presence of *E*-selectin positive bipotent FL hepatoblasts, capable of differentiating into hepatic or biliary epithelial cells (Terrace et al., 2007). Murine hepatoblasts have been characterized by their expression of Protein delta homolog 1 (DLK-1) and a range of important hematopoietic cytokines, such as *Epo, Thpo, Il6, SCF, and Flt3l*. Hepatoblasts also express high levels of ECM molecules, including vitronectin, fibronectin and tenascin C that are expressed under the control of TBGβ1 signaling (Sugiyama et al., 2013). The importance of hepatocytes in expanding HSCs was demonstrated by the co-culture of hepatocyte cell lines with FL cells, which resulted in a large increase in the number of HSCs (Aiuti et al., 1998). Their importance was further underscored by the analysis of *Map2k4*-mutant mouse embryos, lacking hepatoblasts, where hematopoiesis was strongly affected following a decrease in cytokines expression in the FL, such as *EPO* and *SCF*. Accordingly, these embryos displayed a strong decrease in the number of HSCs (Sugiyama et al., 2011).

Another study showed that DLK1-positive hepatocyte progenitors (or hepatoblasts) were the main contributor in the FL niche, as they were the main source of *SCF, IGF-2, Cxcl12* as well as *angptl2* and *angptl3*. Initially, these cells were identified as T cells as they were stained with the anti-CD3 antibody (Chou and Lodish, 2010). *Ex vivo* co-culture of these FL hepatoblasts with BM-HSCs leads to enhanced HSC long-term repopulation and *ex vivo* expansion of HSCs (Zhang and Lodish, 2004; Zhang et al., 2006). It was further suggested that *Angptl2* could mediate its effects through the LILRB2 receptor (Deng et al., 2014).

### Hypoxia and ROS

Hypoxia is an important maturation signal in the microenvironment. In drosophila, low oxygen levels are sensed by Sima, the ortholog of hypoxia-inducible factor 1-α (Hif1-α) that induces crystal cell differentiation. Moderate ROS levels are required for proliferation of early progenitors. Fine-tuning levels of ROS is also required to balance progenitor differentiation in the MZ by regulating *E*-cadherin levels (Gao et al., 2014). Although ROS levels and hypoxia are well-described cues relevant to the adult BM niche, very little is known about their role during embryonic HSC expansion in any other models.

### Transcription Factors Controlling the HSC Niche

Understanding the genetic network that controls the specification and maintenance of the niche will allow building or reproducing these niches *in vitro*. Transcription factors control many aspects of the niche, from how the niche attracts HSCs to their nurturing and release. As mentioned earlier, *klf6a* is a transcription factor that controls the expression of *ccl25b* in cECs from the zebrafish CHT. Therefore, in the absence of *klf6a*, HSCs cannot colonize the CHT and do not expand (Xue et al., 2017).

In the zebrafish as well, we have shown the role of *tfec*, a *mitf* family zinc finger transcription factor (Lister et al., 2011), in HSC expansion. *Tfec* is highly expressed in cECs of the CHT and controls the expression of several CHT niche cytokines (such as *kitlgb*, *thpo*, *csf1a*, and *csf3b*) (Mahony et al., 2016). These cytokines are known to promote HSC proliferation and survival, resulting in their expansion. Consequently, in *tfec* mutants, HSCs fail to mature, resulting in severe anemia (Mahony et al., 2016). However, this phenotype can be rescued by the overexpression of *kitlgb* (Mahony et al., 2016). In addition, *tfec* also controls the expression of *oncostatin M* (*osm)* from cECs that can synergistically enhance the expansion of HSCs with *kitlgb* (Mahony et al., 2018). *Tfec* is therefore a critical transcription factor required for the proper role of the embryonic hematopoietic niche. This role of *tfec* has probably been conserved in mammals. Indeed, in rats, the comparison of liver sinusoidal ECs to lung microvascular ECs revealed a specific combination of transcription factors that controlled liver ECs function and identity (Geraud et al., 2010). *Tfec* was identified as a liver specific transcription factor, together with *Gata4, Lmo3*, and *Maf*. Whereas the roles of *Tfec*, *Lmo3*, and *Maf* have not yet been investigated during mouse fetal hematopoiesis, *Gata4* seems to be required for ECs to function as an HSC niche (Geraud et al., 2017). Further studies demonstrated that *Gata4* is required for correct EC maturation in the embryonic liver and correct HSPC colonization, as *Gata4* allows the specification of a discontinuous endothelium that allows HSC colonization. Therefore, when *Gata4* was specifically deleted in liver sinusoidal ECs during development, the FL vasculature failed to develop normally and hematopoiesis was not supported by the FL (Geraud et al., 2017). The deletion of another transcription factor, *activating transcription factor 4* (*ATF4*) impaired the ability of both endothelial and stromal cells to support FL HSC development. Further analysis revealed that *Atf4* directly regulates the expression of *Angptl3* to control the repopulating efficiency of HSCs (Zhao et al., 2015). Of note, *Atf4* is also required at the cell-autonomous level for proper self-renewal of FL HSCs (Rieger, 2015).

## HSC Release From the Embryonic Niche

The cellular processes governing mammalian HSC release from the embryonic niche remain to be fully characterized. However, a recent study has revealed that the FL-to-BM transition required correct formation of the Wiskott–Aldrich syndrome verprolin-homologous (WAVE) protein complex 2, mediated by Hem-1 (Shao et al., 2018). The disruption of this complex leads to a loss of the survival signal c-Abl in FL-HSCs, premature death and reduced BM colonization, highlighting a cell-intrinsic pathway that is required for FL-to-BM transition.

The HSC migration from FL to the BM is mediated by different cytokines, ECM signals and adhesion molecules (e.g., CXCL12, SCF, cadherins, integrins) (Ciriza et al., 2013). HSCs are then maintained in a quiescent state in BM throughout adulthood by many different signals and cells (Boulais and Frenette, 2015).

The mechanisms driving HSC release from the embryonic niche are similarly elusive in zebrafish. However, live imaging has revealed significant changes in the vascular architecture that normally accommodates HSCs. At 4 dpf, the number of HSCs present in the CHT decreases and they are no longer embedded in the vascular network, but they are instead found loosely associated to vascular cells (Tamplin et al., 2015; Mahony et al., 2016). Further studies have indicated that *mmp9* expression in macrophages was required to modulate the accessibility of HSCs to *cxcl12*. Indeed, the inhibition of *mmp9* resulted in the accumulation of HSCs in the CHT, which was rescued in *cxcl12a*-morphants (Theodore et al., 2017). Therefore the modulation of the vascular structure and the ECM by *mmp9* is an important feature to control retention and release of HSCs from the embryonic niche.

## Evolutionary Conserved Elements

Hematopoietic stem cell emergence and derivation from ECs is a highly conserved process across chick, zebrafish, mouse and humans (Jaffredo et al., 2000; Bollerot et al., 2005a,b; Zovein et al., 2008; Chen et al., 2009; Bertrand et al., 2010; Boisset et al., 2010). Even drosophila blood cells are found in close association to the cardiac tube (Dey et al., 2016). Furthermore, the cell autonomous expression of transcription factors (such as *runx1* and *gata2*) required to achieve EHT is highly conserved. This knowledge gained from the study of different animal models has made it possible to generate inducible HSCs from ECs *in vitro* (Gomes et al., 2018).

Across the species discussed in this review there are several conserved processes but also many differences in how HSCs expand in their fetal niche. Understanding the main similarities and differences is crucial to fully understanding HSC expansion. Here we will summarize the main conserved elements across drosophila, zebrafish, mouse and human and how these have progressed throughout evolution.

**FIGURE 1:**
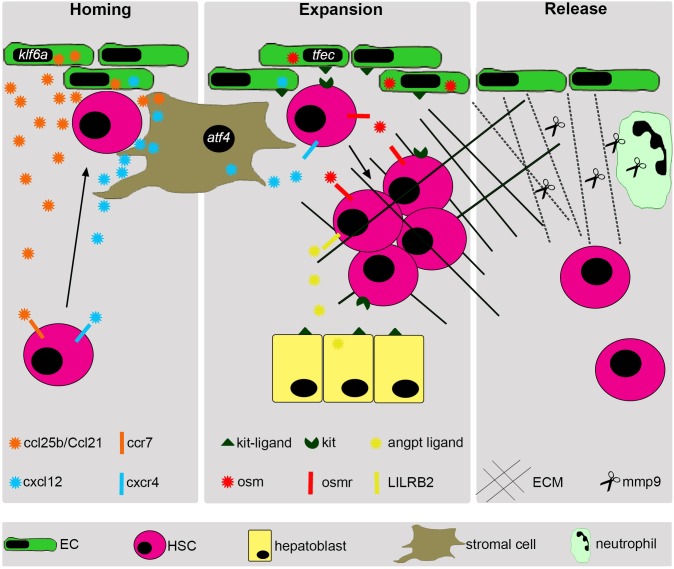
Summary of vertebrate HSC expansion in the embryonic niche. Following their derivation from aortic endothelium, HSCs home to their embryonic niche in response to several attractive cytokines, such as *cxcl12* and *ccl25b/Ccl21*. HSCs are directed toward vascular cells by Mφ (macrophages). The vascular cells in the embryonic niche then remodel to accommodate the arriving HSCs. HSCs then become lodged in the fetal niche and undergo cell division to expand their initial number. This expansion is in response to several cytokines released from many different cell types. Endothelial cells release *kitlg* and *osm*, under the control of *tfec*. Stromal cells release *cxcl12*, under the control of *atf4*. Hepatoblasts release *Kit-ligand* and angiopoietins. After considerable expansion, the ECM is remodeled by *Mmp9* released from neutrophils and HSCs leave their fetal niche to migrate to their adult niche. Outlined here is an overview of the main cell types involved in fetal HSC expansion along with some examples of important cytokines/signals that they secrete, although many others exist.

The drosophila PSC consists of stromal cells in the lymph gland, and controls blood cell production and maturation (Lebestky et al., 2003). These cells are responsible for the signaling of a number of extrinsic factors to control HSC expansion (Figure 1 and Table 2). Throughout evolution, the structure of the embryonic niche has greatly changed. The vertebrate phylum coincides with the emergence of a closed circulatory system, dependent on vasculature. In the zebrafish, the role of vasculature becomes very important for HSC expansion (Tamplin et al., 2015) and remains so in higher vertebrate species (Table 2). In avian embryos, HSCs expand in the yolk sac, a highly vascularized structure (Guedes et al., 2014), and mouse HSCs are also closely associated to the growing vasculature in the FL that support HSCs (Ohneda and Bautch, 1997; Wittig et al., 2010; Tamplin et al., 2015) (Figure 1 and Table 2).

**Table 2:** Summary of the important cells and tissues required to mediate HSC expansion through evolution.

	Drosophila (Lymph gland)	Zebrafish (CHT)	Xenopus (FL)	Chicken (PAF +YS)	Mammals (FL)
HSC Progeny	Yes	?	?	?	?
Stromal cells	Yes	Yes	Yes	Yes	Yes
Endothelial cells	No	Yes	Yes	Yes (YS)	Yes
Hepatocytes	No	No	?	No	Yes
Nervous system	?	?	?	?	?

*CHT, caudal hematopoietic tissue; FL, fetal liver; PAF, para-aortic foci; YS, yolk sac.*

From zebrafish to mammals, HSCs expand in an increasingly more complex niche: whereas the CHT in teleosts is a transient vascularized tissue (Tamplin et al., 2015), HSCs will colonize the FL in mammals, a *bona fide* organ. With this, comes the addition of a new cellular layer to the HSC niche, i.e., the hepatocytes. As stromal and ECs, hepatoblasts will secrete similar survival and proliferative signals, such as SCF and other cytokines (Sugiyama et al., 2011, 2013) (Figure 1 and Table 2). It is interesting to note that even if the structures have changed, the genetic network seems to have been conserved. One such example is the transcription factor *Tfec* that is specifically expressed in the zebrafish CHT vasculature, as well as in sinusoidal ECs of the fetal and adult liver in rodents (Geraud et al., 2010; Mahony et al., 2016).

## Clinical Implications

The recent use of drug screens has identified several promising candidates to improve the clinical use of HSCs in regenerative medicine. For example, StemRegenin-1 (SR1), an antagonist of the aryl hydrocarbon receptor (AHR) enhances the *ex vivo* expansion of CD34^+^ cells and improves long-term engraftment in murine models (Boitano et al., 2010). Recently, early phase clinical trials have further highlighted the effectiveness of this compound in improving the expansion of CD34^+^ cells from umbilical cord blood (Wagner et al., 2016). UM171, another compound, improves human cord blood cell expansion and engraftment, although the exact mechanism remains to be fully characterized (Fares et al., 2014). Zebrafish high throughput screens have also highlighted the role of dmPGE2 that confers a competitive advantage to treated HSCs and has been successful in subsequent clinical trials (North et al., 2007; Cutler et al., 2013; Goessling and North, 2014). Identifying additional compounds and combinations of cytokines will improve HSC expansion for therapeutic use.

The importance in further understanding the niche has been further underscored as it was shown that niche dysfunctions (for example, induced by mesenchymal infections) could lead to genotoxic stress and might sensitize HSCs to leukemia development (Zambetti et al., 2016; Passaro et al., 2017). It remains to be identified if aberrant cytokine signaling in the fetal niche can sensitize HSCs to different disorders.

## Conclusion

Understanding the complete genetic and molecular program that controls HSC expansion in the embryonic niche remains an important goal to improve current protocols of regenerative medicine. The use of many animal models across phylogeny will concur to this aim, as most of the mechanisms involved in the control of HSC expansion by the embryonic niche appear to be conserved through evolution (Figure 1 and Table 2).

## Author Contributions

CBM wrote the manuscript. JYB edited the manuscript.

## Conflict of Interest Statement

The authors declare that the research was conducted in the absence of any commercial or financial relationships that could be construed as a potential conflict of interest.
